# On the Secure Performance of Intelligent Reflecting Surface-Assisted HARQ Systems

**DOI:** 10.3390/e25030519

**Published:** 2023-03-17

**Authors:** Yue Wu, Kuanlin Mu, Kaiyu Duan, Shishu Yin, Hongwen Yang

**Affiliations:** 1 Department of Electronic and Information Engineering, Anhui University of Finance and Economics, Bengbu 233030, China; 2 School of Information and Communication Engineering, Beijing University of Posts and Telecommunications, Beijing 100876, China

**Keywords:** chase combining (CC), hybrid automatic repeat request (HARQ), incremental redundancy (IR), intelligent reflecting surface (IRS), physical layer security (PLS)

## Abstract

This paper analyzes the physical layer security performance of hybrid automatic repeat request (HARQ) systems with the assistance of an intelligent reflecting surface (IRS) and aims to reveal the primary factors that enhance PLS. First, closed-form expressions for the connection outage probability (COP) and secrecy outage probability (SOP) in HARQ with chase combining (HARQ-CC) are acquired using the generalized-*K* (KG) distribution. Then, these two critical metrics are derived while adopting HARQ with incremental redundancy (HARQ-IR), resorting to the mixture gamma (MG) distribution and the Mellin transform. Diversity and coding gain are also addressed through an asymptotic analysis of the COP and SOP. Finally, an evaluation of the numerical results demonstrates that a greater gain in the main channel and the wiretap channel can be produced by increasing the number of meta-surfaces rather than increasing the maximum transmission number, except for the higher signal-to-noise (SNR) region of HARQ-IR where the latter is preferred. This finding provides a significant guidance for the joint configuration of IRS and HARQ to achieve secure communication.

## 1. Introduction

Rapid developments in wireless communications have made intelligent reflecting surface (IRS), sometimes referred to as reconfigurable intelligent surface (RIS), an important candidate technology for 6G due to its flexible control and superior performance. IRS can be deployed in different locations with extremely low costs compared to base stations (BSs) or small cells. Smart radio environments (SREs) can also be achieved by adjusting the phase, frequency or other parameters of the reflected signal using a soft-defined architecture, which effectively enhances signal coverage and network transmission performance [1,2].

Significant research has been conducted to speed up the application of IRS, including studies on channel modeling, performance analysis and resource allocation. Generally, IRS channel modeling consists of two components; those are the transmitter-IRS channel and the IRS-receiver channel. A typical approach is to uniformly model the transmitter-IRS-receiver process as a cascade channel (i.e., the product of a two-part channel fading [3,4,5]). Basar et al. proposed a phase adjusting scheme aiming to improve the received signal-to-noise ratio (SNR) [6]. The central limit theorem (CLT) was applied so as to assess the probability density function (PDF) for this SNR when the amount of meta-surfaces (*N*) was great, to further derive a symbol error rate (SER) under different modulation conditions. However, in some scenarios, it is not necessary to allocate multiple meta-surfaces to a single user, and thus CLT is not suitable when *N* is small. Yang et al. noted a cascade channel can be accurately modeled with the generalized-*K* (KG) distribution [7]. An analysis conducted by Zhang et al. further demonstrated the influence of limited phase shifts on achievable performance levels [8]. Outage probability and spectral efficiency have also been deduced in multiple-element IRS-assisted systems [9]. In addition, Boulogeorgos et al. compared the outage probability and ergodic capacity of IRS-assisted and amplify-and-forward (AF) relaying wireless systems [10]. Huang et al. maximized spectral and energy efficiency through transmitting power allocation and meta-surface phase shifts [11]. Guo and Yang et al. optimized the achievable rate through dynamic passive beamforming in an IRS-assisted system while considering orthogonal frequency division multiplexing (OFDM) and multi-user channels [12,13].

Physical layer security (PLS), which ensures secure performance through a wireless propagation environment, has received increased attention recently. This is in part due to IRS systems that can flexibly configure a wireless propagation channel with the assistance of IRS. Connection outage probability (COP), secrecy outage probability (SOP) and secrecy rate (i.e., secrecy capacity) are commonly used performance optimization metrics for these systems. COP mainly describes the likelihood that the legitimate receiver (Bob) cannot decode transmitted codewords, and SOP gives the possibility that the eavesdropper (Eve) cannot be confused by secrecy redundancy after the k-th transmission. They indicate the reliability and security, respectively, while secrecy rate shows the overall secure performance [14,15]. For example, Yang et al. developed a closed-form solution of SOP and identified a corresponding asymptotic value in an IRS-assisted communication system using CLT [16]. Khoshafa et al. proposed an SOP for an IRS-assisted D2D system involving Meijer’s G-function [17]. Shen et al. investigated secrecy rate maximization for multi-antenna IRS systems by combined optimum design of source transmission covariance and IRS phase shift matrices [18]. This joint optimization was also achieved by Cui et al., who solved the problem through beamforming to enhance the secrecy rate [19] while taking into account a complex scenario where the wiretap channel exceeded the main channel. Similarly, Hong et al. applied a covariance matrix of artificial noise (AN) to the joint optimization of a block coordinate descent (BCD) algorithm [20]. This was done to maximize the secrecy rate when the transmitting power limit and unit modulus of the IRS phase shifts were constrained. A fast solution was proposed to solve these constrained optimization problems with a modified genetic algorithm [21]. Yu et al. introduced a robust secure transmission scheme, which considered imperfect channel state information (CSI) in the wiretap channel [22]. This system was designed to improve the sum-rate capacity with constrained highest information leakage for possible eavesdroppers. Wang et al. proposed a joint beamforming and jamming strategy that improved security in a more realistic scenario, in which the CSI was absent [23]. Gu et al. expressed the probabilities of a nonzero secrecy capacity and an ergodic secrecy capacity using closed-form expressions while considering the distribution of eavesdropper locations [24].

As a classical error-control technology, HARQ simultaneously ensures reliability and effectiveness using re-transmission and combining mechanisms. Previous studies have suggested that HARQ is also capable of improving physical layer security. Typical HARQ schemes primarily include chase combining (CC) and incremental redundancy (IR). In the case of HARQ-CC, decoding errors in the receiver trigger the re-transmission of same redundancy versions. Soft information from initial transmissions and re-transmissions is then combined, producing gains in the SNR. Unlike HARQ-CC, HARQ-IR constructs re-transmitted data using various redundancy versions, thus achieving higher coding gain. This approach is not only more efficient, it is also more complex and improvements to its design have been the subject of several recent studies [25,26,27]. In addition, Tang et al. gave the discussion of secrecy outage probability and secrecy throughput in secure HARQ transmissions [28]. Mheich et al. designed a rate adaptive secure HARQ scheme [29], and Guan et al. addressed the reliability–security trade-off and accurate secrecy throughput [30]. It has been demonstrated that an eavesdropper cannot obtain the same diversity gain as a legitimate user, since re-transmissions rely solely on the latter user [31]. As another import metric, effective secrecy throughput (EST) with multiple eavesdroppers has been optimized in secure HARQ transmissions [32]. Additionally, Park proposed Kalman combining-based iterative detection and decoding schemes for multiple-input multiple-output (MIMO) systems with HARQ [33], and Wu et al. achieved a tradeoff between timeliness and reliability for HARQ-CC aided non-orthogonal multiple-access (NOMA) systems [34].

HARQ plays an important role in error-control mechanisms for IRS-assisted systems, as its diversity attributes will inevitably impact reliability and security. Cao et al. addressed the outage rate and progressiveness of Type-1 and HARQ-CC for multi-RIS systems [35]. However, a similar study has yet to be conducted for the more efficient HARQ-IR scheme. Moreover, the performance of PLS in IRS-assisted HARQ systems has yet to be reported. As such, this study first analyzes secure IRS-assisted HARQ performance. Additional contributions of this work include the following:

- Closed-form expressions for COP and SOP are derived in IRS-assisted HARQ-CC systems while considering the differences in received SNR between the main channel and the wiretap channel. These two SNR distributions can be accurately approximated by the sum of KG distributed random variables (RVs) with varying parameters.
- Closed-form expressions for COP and SOP in IRS-assisted HARQ-IR systems are derived with the stated differences in received SNR. The mixture gamma (MG) distribution and the Mellin transform are then required to efficiently solve this problem, owing to the computing complexity of IR.
- Diversity gain and coding gain in the main channel and wiretap channel are then derived via COP/SOP and their asymptotic values using a series expansion of Meijer’s G-function.
- Numerical results verify the accuracy of our derivations and confirm that the amounts of meta-surfaces and the maximum transmission number have varying influence on COP and SOP for HARQ-CC and HARQ-IR.

The remainder of this paper is structured as follows. Section 2 describes the overall system model as well as secure transmission without HARQ. Section 3 derives closed-form expressions for COP and SOP in IRS-assisted systems with HARQ-CC and HARQ-IR, respectively. Section 4 provides asymptotic values for COP and SOP, along with their corresponding diversity gain and coding gain. Numerical and simulated results are discussed in Section 5. Section 6 concisely concludes our study.

Notation: $K_{v}\left(\cdot \right)$ represents the modified Bessel function of the second kind and order *v*, Γ(·) denotes the gamma function, E[·] indicates the expectation operator, $G_{p,q}^{m,n}\left[\cdot \right]$ is Meijer’s G-function [36], ${\left(\cdot \right)}^{T}$ indicates the transpose of a matrix, and R and Z represent the sets of real numbers and integers, respectively.

## 2. System Model for IRS-Assisted Secure HARQ Transmissions

### 2.1. System Model

**Figure 1:**
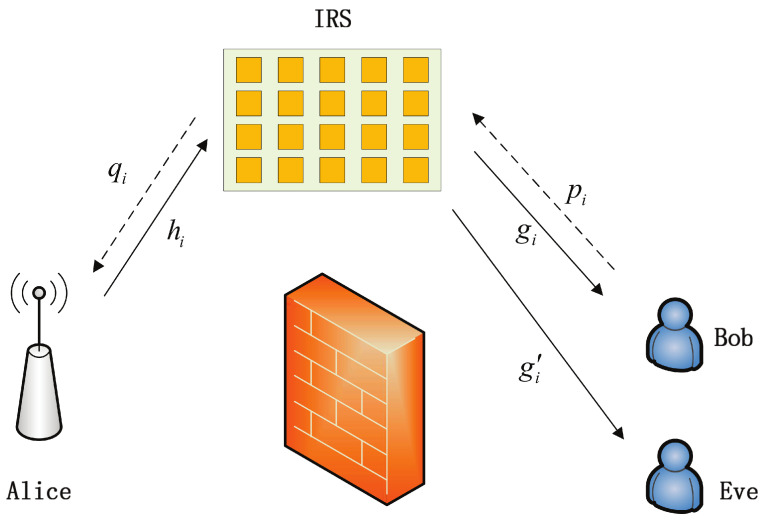
System model of secure IRS-assisted HARQ system.

We take into account an IRS-assisted secure HARQ transmission model, as depicted in Figure 1. In this scenario, an obstacle is positioned between the transmitter (Alice) and the legitimate receiver (Bob). The transmitted signal reaches Bob after being reflected by the IRS while the passive eavesdropper (Eve) intercepts signals from the IRS. These two components of the cascade channel (i.e., from Alice to the IRS and from the IRS to Bob/Eve) are both assumed to be Rayleigh fading channels. The Wyner secrecy code [37] was used for secrecy encoding, with a code rate and secrecy redundancy rate of $R_{B}$ and $R_{E}$, respectively. In the event of erroneous decoding by Bob, he will transmit a negative acknowledgment (NAK) message to Alice, requesting a re-transmission. Otherwise, an acknowledgment (ACK) message is provided for the new transmission, where a re-transmitted code word is constructed using redundancy versions based on HARQ-CC/IR protocols. Re-transmission ceases once a maximum transmission number *K* is reached. In this process, Eve achieves limited gain through multiple signal versions, since re-transmission relies only on decoding results from Bob.

### 2.2. Secure Transmissions without HARQ

We first analyze secure transmissions without re-transmission. In this process, power scaling laws [38] were employed to fix the transmitting power for a single meta-surface path to *P*. The corresponding transmitted symbol *x* then satisfies $E\left[{\left|x\right|}^{2}\right]=P$, where channel fading items for the *i*-th meta-surface from Alice to the IRS and from the IRS to Bob are represented by $h_{i}$ and $g_{i}$, respectively. These paths constitute a cascade main channel with both symmetric complex Gaussian distribution (i.e., $h_{i}∼\mathrm{CN}\left(0,1\right)$ and $g_{i}∼\mathrm{CN}\left(0,1\right)$). In the following sections, the shifted IRS phase is denoted by $\phi_{i}$, $n_{B}$ is additive Gaussian white noise (AWGN) for the whole cascade main channel and $n_{B}∼\mathrm{CN}\left(0,\sigma_{B}^{2}\right)$. The magnitude of the received signal in the main channel can then be expressed as

(1) $$r_{B}=\left[\sum_{i=1}^{N}h_{i}e^{j\phi_{i}}g_{i}\right]x+n_{B}.$$

These two channel fading terms can be represented using complex signals as $h_{i}=\alpha_{i}e^{-j\theta_{i}}$ and $g_{j}=\beta_{i}e^{-j\psi_{i}}$, where $\alpha_{i}$ and $\beta_{i}$ are the corresponding magnitude terms and $\theta_{i}$ and $\psi_{i}$ are the corresponding phase terms. The received SNR of the main channel is

(2) $$\gamma_{B}=\frac{P}{\sigma_{B}^{2}}{\left|\sum_{i=1}^{N}\alpha_{i}\beta_{i}e^{j\left(\phi_{i}-\theta_{i}-\psi_{i}\right)}\right|}^{2}.$$

This SNR is maximized when $\phi_{i}=\theta_{i}+\psi_{i}$ [6], such that

(3) $$\gamma_{B}=\frac{P}{\sigma_{B}^{2}}{\left(\sum_{i=1}^{N}\alpha_{i}\beta_{i}\right)}^{2}={\bar{\gamma }}_{B}R_{1}^{2},$$

where ${\bar{\gamma }}_{B}$ denotes the average received SNR of the main channel and $X_{i}=\alpha_{i}\beta_{i}$. Since the cascade channel is double Rayleigh distributed, $X_{i}$ is also generalized-*K* (KG) distributed. In addition, $R_{1}=\sum_{i=1}^{N}X_{i}$ is the sum of KG distributed RVs approximated using $\sqrt{W}$, where $W=\sum_{i=1}^{N}X_{i}^{2}$ is accurately approximated by a squared KG distribution [7,39]. Hence, the probability density function (PDF) of $R_{1}$ can be obtained by

(4) $$f_{R_{1}}\left(r\right)=\frac{4\Xi_{m}^{k_{m}+m_{m}}}{\Gamma \left(k_{m}\right)\Gamma \left(m_{m}\right)}r^{k_{m}+m_{m}-1}K_{k_{m}-m_{m}}\left(2\Xi_{m}r\right),$$

where $k_{m}$ and $m_{m}$ are shaping parameters, $k_{m}=\frac{-b+\sqrt{b^{2}-4ac}}{2a^{2}}$, and $\Xi_{m}=\sqrt{\frac{k_{m}m_{m}}{\Omega_{m}}}$, where $\Omega_{m}$ is the mean power of $R_{1}$ which can be obtained by its second moment denoted by $\mu_{R_{1}}\left(2\right)$. The calculation of a,b,c and $\mu_{R_{1}}\left(n\right)$ can be found in [39]. The PDF of $\gamma_{B}$ can be expressed as

(5) $$f_{\gamma_{B}}\left(\gamma \right)=\frac{2{\tilde{\Xi }}_{m}^{k_{m}+m_{m}}}{\Gamma \left(k_{m}\right)\Gamma \left(m_{m}\right)}\gamma^{\frac{k_{m}+m_{m}}{2}-1}K_{k_{m}-m_{m}}\left(2{\tilde{\Xi }}_{m}\sqrt{\gamma }\right),$$

where ${\tilde{\Xi }}_{m}=\sqrt{\frac{k_{m}m_{m}}{{\bar{\gamma }}_{B}\Omega_{m}}}$. The closed-form expression of the corresponding cumulative distribution function (CDF) can be acquired from $F_{\gamma_{B}}\left(\gamma \right)=\int_{0}^{\infty }f_{\gamma_{B}}\left(\gamma \right)d\gamma$ as

(6) $$F_{\gamma_{B}}\left(\gamma \right)=\frac{1}{\Gamma \left(k_{w}\right)\Gamma \left(m_{w}\right)}G_{1,3}^{2,1}\left({\tilde{\Xi }}_{m}^{2}\gamma |\begin{array}{c} 1\\ k_{w},m_{w},0 \end{array}\right).$$

In the case of the wiretap channel, which is the cascade channel constructed from components of Alice-IRS and IRS-Eve, channel fading and phase adjustments from Alice to IRS are consistent with the main channel. In other words, $h_{i}$ and $\phi_{i}$ are defined as shown above. The other part of channel fading from IRS to Eve is indicated by $g_{i}^{\prime }$ and thus $g_{i}^{\prime }∼\mathrm{CN}\left(0,1\right)$, where $n_{E}$ is the AWGN of the whole cascade wiretap channel and $n_{E}∼\mathrm{CN}\left(0,\sigma_{E}^{2}\right)$. The received signal in the wiretap channel is then given by

(7) $$r_{E}=\left[\sum_{i=1}^{N}h_{i}e^{j\phi_{i}}g_{i}^{\prime }\right]x+n_{E}.$$

After adjusting $\phi_{i}$ given in the main channel, $h_{i}e^{j\phi_{i}}$ and $h_{i}$ follow the same distribution due to the characteristics of a cyclic symmetric complex Gaussian distribution. Assuming $h_{i}^{*}=h_{i}e^{j\phi_{i}}$, the received SNR of the wiretap channel is given by

(8) $$\gamma_{E}=\frac{P}{\sigma_{E}^{2}}{\left(\sum_{i=1}^{N}h_{i}^{*}g_{i}^{\prime }\right)}^{2}={\bar{\gamma }}_{E}\left({\left(h_{i}^{*}\right)}^{T}\cdot g_{i}^{\prime }\right).$$

The PDF of the cascaded channel amplitude is known as a KG distributed model [40] and the corresponding CDF follows a squared KG distribution. Hence, the PDF of $\gamma_{E}$ is given by

(9) $$f_{\gamma_{E}}\left(\gamma \right)=\frac{2{\tilde{\Xi }}_{w}^{N+1}\gamma^{\frac{N-1}{2}}}{\Gamma (N)}K_{N-1}\left(2{\tilde{\Xi }}_{w}\sqrt{\gamma }\right),$$

where ${\tilde{\Xi }}_{w}=\sqrt{\frac{1}{{\bar{\gamma }}_{E}}}$, $K_{v}\left(x\right)$ is a modified Bessel function of the second kind (order *v*) and $k_{w}=N$ and $m_{w}=1$ are shaping parameters. In addition, $\Xi_{w}=\sqrt{\frac{k_{w}m_{w}}{\Omega_{w}}}=1$, where $\Omega_{w}=N$. The CDF of $\gamma_{E}$ is

(10) $$F_{\gamma_{E}}\left(\gamma \right)=\frac{1}{\Gamma (N)}G_{1,3}^{2,1}\left({\tilde{\Xi }}_{w}^{2}\gamma |\begin{array}{c} 1\\ N,1,0 \end{array}\right).$$

## 3. Outage Probability of IRS-Assisted Secure HARQ

In this section, we discuss the outage probability (OP) performance of IRS-assisted secure HARQ communication systems, involving connection outage probability (COP) and secrecy outage probability (SOP). These two metrics are derived in HARQ-CC and HARQ-IR, respectively, and are used to evaluate the critical secure performance.

### 3.1. OP of IRS-Assisted Secure HARQ-CC

The HARQ-CC case is first analyzed, in which a connection outage occurs if less mutual information of the main channel is accumulated compared with $R_{B}$. This corresponding COP is given by

(11) $$P_{e}^{CC}=\mathrm{Pr}\left\{I_{B}^{CC}\left(K\right)<R_{B}\right\},$$

where $I_{B}^{CC}\left(K\right)$ denotes the accumulated mutual information after the *K*-th transmission. When adopting maximum ratio combining (MRC), $I_{B}^{CC}\left(K\right)=\log_{2}\left(1+\sum_{k=1}^{K}\gamma_{B,k}\right)$, where $\gamma_{B,k}$ is the received SNR of the *k*-th transmission in the main channel. This term exhibits a KG distribution and its PDF can be expressed by Equation (5). Assuming $\Upsilon_{B,K}=\sum_{k=1}^{K}\gamma_{B,k}$ implies

(12) $$P_{e}^{CC}=\mathrm{Pr}\left\{\Upsilon_{B,K}<2^{R_{B}}-1\right\}=F_{\Upsilon_{B,K}}\left(2^{R_{B}}-1\right).$$

The sum of multiple KG-distributed RVs can be accurately approximated by a squared KG distribution [41]. The corresponding PDF is thus

(13) $$f_{\Upsilon_{B,K}}\left(\gamma \right)=\frac{2{\tilde{\Xi }}_{M}^{k_{M}+m_{M}}\gamma^{\frac{k_{M}+m_{M}}{2}-1}}{\Gamma \left(k_{M}\right)\Gamma \left(m_{M}\right)}K_{k_{M}-m_{M}}\left(2{\tilde{\Xi }}_{M}\sqrt{\gamma }\right),$$

where the fading parameters are $k_{M}=Kk_{m}$ and $m_{M}=Km_{m}$. Additionally, ${\tilde{\Xi }}_{M}=\sqrt{\frac{k_{M}m_{M}}{{\bar{\gamma }}_{B}\Omega_{M}}}$, where $\Omega_{M}=K\Omega_{m}$. After integrating the above formula, the CDF of $\Upsilon_{B,K}$ can be obtained by

(14) $$F_{\Upsilon_{B,K}}\left(\gamma \right)=\frac{1}{\Gamma \left(k_{M}\right)\Gamma \left(m_{M}\right)}G_{1,3}^{2,1}\left({\tilde{\Xi }}_{M}^{2}\gamma |\begin{array}{c} 1\\ k_{M},m_{M},0 \end{array}\right).$$

The COP of HARQ-CC can be acquired by instituting $F_{\Upsilon_{B,K}}\left(\gamma \right)$ in Equation (12). The probability of the *k*-th transmission is then given by

(15) $$\begin{array}{cc} \mathrm{Pr}\{M=k\}\; & =P_{e}^{CC}\left(k-1\right)-P_{e}^{CC}\left(k\right)\\ \; & =F_{\Upsilon_{B,k-1}}\left(2^{R_{B}}-1\right)-F_{\Upsilon_{B,k}}\left(2^{R_{B}}-1\right), \end{array}$$

where *M* denotes the actual transmission number and $P_{e}^{CC}\left(0\right)=1$. Hence, the average transmission number of HARQ-CC is

(16) $$\begin{array}{cc} \bar{M} & =E\left(M\right)=\sum_{k=1}^{K}k\mathrm{Pr}\left\{M=k\right\}\\ \; & =1+\sum_{k=1}^{K-1}F_{\Upsilon_{B,K}}\left(2^{R_{B}}-1\right). \end{array}$$

Since a secrecy outage occurs when the mutual information is greater than the secrecy redundancy rate in the wiretap channel, the SOP of HARQ-CC after the *k*-th transmission can be expressed as

(17) $$\begin{array}{cc} \mathrm{Pr}\{I_{E}^{CC}\left(K\right)\geq R_{E}\}\; & =\mathrm{Pr}\left\{\log_{2}\left(1+\sum_{k=1}^{K}\gamma_{E,k}\right)\geq R_{E}\right\}\\ \; & =1-F_{\Upsilon_{E,K}}\left(2^{R_{E}}-1\right), \end{array}$$

where $I_{E}^{CC}\left(K\right)$ represents the wiretap channel mutual information accumulated by HARQ-CC after the *K*-th transmission and $\gamma_{E,k}$ denotes the received SNR at Eve during the *k*-th transmission, with a distribution given by Equation (9). We assume $\Upsilon_{E,K}=\sum_{k=1}^{K}\gamma_{E,k}$ and it has been already demonstrated that the sum of the aforementioned KG distributed RVs still follows a KG distribution with relevant parameters. The corresponding PDF can be represented by

(18) $$f_{\Upsilon_{E,K}}\left(\gamma \right)=\frac{2{\tilde{\Xi }}_{W}^{k_{W}+m_{W}}\gamma^{\frac{k_{W}+m_{W}}{2}-1}}{\Gamma \left(k_{W}\right)\Gamma \left(m_{W}\right)}K_{k_{W}-m_{W}}\left(2{\tilde{\Xi }}_{W}\sqrt{\gamma }\right),$$

where $k_{W}=KN$ and $m_{W}=K$ are shaping parameters and ${\tilde{\Xi }}_{W}=\sqrt{K}{\tilde{\Xi }}_{w}$. The CDF of $\Upsilon_{E,K}$ is then given by

(19) $$F_{\Upsilon_{E,K}}\left(\gamma \right)=\frac{1}{\Gamma (KN)\Gamma (K)}G_{1,3}^{2,1}\left(K{\tilde{\Xi }}_{w}^{2}\gamma |\begin{array}{c} 1\\ KN,K,0 \end{array}\right).$$

Since the actual transmission number *M* is solely dependent on the main channel, the SOP of HARQ-CC can be expressed as

(20) $$\begin{array}{cc} P_{s}^{CC}\; & =\sum_{k=1}^{K}\mathrm{Pr}\left\{M=k\right\}\mathrm{Pr}\left\{I_{E}^{CC}\left(k\right)\geq R_{E}\right\}\\ \; & =\sum_{k=1}^{K}\left(F_{\Upsilon_{B,k-1}}\left(2^{R_{B}}-1\right)-F_{\Upsilon_{B,k}}\left(2^{R_{B}}-1\right)\right)\left(1-F_{\Upsilon_{E,k}}\left(2^{R_{E}}-1\right)\right), \end{array}$$

where $F_{\Upsilon_{B,k}}\left(\gamma \right)$ and $F_{\Upsilon_{E,K}}\left(\gamma \right)$ are defined by Equations (14) and (19), respectively.

### 3.2. OP of IRS-Assisted Secure HARQ-IR

Unlike HARQ-CC, HARQ-IR forms code words using different redundancy versions, rather than the same re-transmitted data. After soft-combining, mutual information (not SNR) is directly accumulated. For the main channel, the COP of HARQ-IR is thus

(21) $$P_{e}^{IR}=\mathrm{Pr}\left\{I_{B}^{IR}\left(K\right)<R_{B}\right\},$$

where accumulated mutual information after the *K*-th transmissions is given by

(22) $$\begin{array}{cc} I_{B}^{IR}\left(K\right) & =\sum_{k=1}^{K}\log_{2}\left(1+\gamma_{B,k}\right)\\ \; & =\log_{2}\prod_{k=1}^{K}\left(1+\gamma_{B,k}\right). \end{array}$$

We can assume $Z_{B,K}=\prod_{k=1}^{K}Y_{B,k}$ and $Y_{B,k}=1+\gamma_{B,k}$, where $Y_{B,k}$ is a shifted squared KG distributed RV and $\gamma_{B,k}$ is given in Equation (5). Thus

(23) $$\begin{array}{cc} P_{e}^{IR}\; & =\mathrm{Pr}\left\{\prod_{k=1}^{K}Y_{B,k}<2^{R_{B}}\right\}\\ \; & =\mathrm{Pr}\left\{Z_{B,k}<2^{R_{B}}\right\}\\ \; & =F_{Z_{B,K}}\left(2^{R_{B}}\right), \end{array}$$

where the CDF of $Z_{B,K}$ is denoted by $F_{Z_{B,K}}\left(\cdot \right)$ and $\gamma_{B,k}$ follows a squared KG distribution. However, the PDF and CDF of $Z_{B,K}$ are difficult to determine. Therefore, we resort to applying the mixture gamma (MG) distribution [42] to express the PDF of $\gamma_{B,k}$ as

(24) $$f_{\gamma_{B,k}}\left(\gamma \right)=\sum_{i=1}^{L}\alpha_{m,i}\gamma^{\beta_{m,i}-1}e^{-\zeta_{m,i}\gamma },\gamma \geq 0,$$

where $\alpha_{m,i}=\psi \left(\theta_{m,i},\beta_{m,i},\zeta_{m,i}\right)=\frac{\theta_{m,i}}{\sum_{j=1}^{L}\theta_{m,j}\Gamma \left(\beta_{m,j}\right)\zeta_{m,j}^{-\beta_{m,j}}}$, $\beta_{m,i}=m_{m}$, $\zeta_{m,i}=\frac{\lambda_{m}}{t_{m,i}}$, $\lambda_{m}={\tilde{\Xi }}_{m}^{2}$, $\theta_{m,i}=\frac{\lambda^{m_{m}}\omega_{i}t^{k_{m}-m_{m}-1}}{\Gamma \left(k_{m}\right)\Gamma \left(m_{m}\right)}$. The amount of summation terms in this distribution is denoted by *L*, while $\omega_{i}$ and $t_{i}$ are the weight and abscissas factors in the Gaussian–Laguerre integration [43]. The $k_{m}$, $m_{m}$ and ${\tilde{\Xi }}_{m}$ were defined in Equation (5). Furthermore, $Y_{B,k}$ can be expressed as a combination of the shifted Gamma distributed RVs, with a PDF given by

(25) $$f_{Y_{B,k}}\left(y\right)=\sum_{i=1}^{L}\alpha_{m,i}{\left(y-1\right)}^{\beta_{m,i}-1}e^{-\zeta_{m,i}\left(y-1\right)},y\geq 1.$$

A Mellin transform of $f_{Y_{B,k}}\left(y\right)$ can then be used to derive the distribution of $Z_{B,K}$ as

(26) $$\begin{array}{cc} M_{s}\left\{f_{Y_{B,k}}\left(y\right)\right\}= & \int_{1}^{\infty }y^{s-1}f_{Y_{B,k}}\left(y\right)dy\\ = & \int_{1}^{\infty }y^{s-1}\sum_{i=1}^{L}\alpha_{m,i}{\left(y-1\right)}^{\beta_{m,i}-1}e^{-\zeta_{m,i}\left(y-1\right)}dy\\ = & \sum_{i=1}^{L}\int_{0}^{\infty }{\left(1+t\right)}^{s-1}\alpha_{m,i}t^{\beta_{m,i}-1}e^{-\zeta_{m,i}t}dt\\ = & \sum_{i=1}^{L}\alpha_{i}\Gamma \left(\beta_{m,i}\right)\Psi \left(\beta_{m,i},\beta_{m,i}+s;\zeta_{m,i}\right), \end{array}$$

where $\Psi \left(\alpha ,\gamma ;z\right)=\frac{1}{\Gamma (\alpha )}\int_{0}^{\infty }e^{-zt}t^{\alpha -1}{\left(1+t\right)}^{\gamma -\alpha -1}dt$ denotes Tricomi’s confluent hyper-geometric function [36].

According to the properties of Mellin transform [44],

(27) MsfZB,K(z)=∏k=1KMsfYB,k(y)=∑i=1Lαm,iΓ(βm,i)Ψ(βm,i,βm,i+s;ζm,i)K=∑r1⋯rLr1+⋯+rL=KKr1⋯rL∏j=1Lαm,jΓ(βm,j)Ψ(βm,j,βm,j+s;ζm,j)rj,

where Kr1⋯rL=K!r1!⋯rp! and the last equation holds by the polynomial theorem. The inverse Mellin transform of $M_{s}\left\{f_{Z_{B,K}}\left(z\right)\right\}$ is provided in Appendix A and the PDF $f_{Z_{B,K}}\left(z\right)$ is shown as

(28) fZB,K(z)=∑r1⋯rLr1+⋯+rL=KKr1⋯rL∏j=1Lαm,jΓ(βm,j)ζm,j−βm,j+1rj×Y0,KK,0z∏j=1Lζm,jrj|−(0,1,ζm,1,βm,1),⋯,(0,1,ζm,1,βm,1)︸r1items,⋯,(0,1,ζm,L,βm,L),⋯,(0,1,ζm,L,βm,L)︸rLitems

The CDF of $Z_{B,K}$ can be deduced by integrating the PDF above [45] as

(29) FZB,K(z)=∑r1⋯rLr1+⋯+rL=KKr1⋯rL∏j=1Lαm,jΓ(βm,j)ζm,j−βm,jrj×1−Y1,K+1K+1,0z∏j=1Lζm,jrj|(1,1,0,1)(0,1,0,1),(0,1,ζm,1,βm,1),⋯,(0,1,ζm,1,βm,1)︸r1items,⋯,(0,1,ζm,L,βm,L),⋯,(0,1,ζm,L,βm,L)︸rLitems

As a result, the COP of HARQ-IR becomes $F_{Z_{B,K}}\left(2^{R_{B}}\right)$. The average transmission number of HARQ-IR is then

(30) $${\bar{M}}^{\prime }=1+\sum_{k=1}^{K-1}F_{Z_{B,k}}\left(2^{R_{B}}\right),$$

and the probability of a secrecy outage occurring after the *K*-th transmission is

(31) $$\mathrm{Pr}\left\{I_{E}^{IR}\left(K\right)\geq R_{E}\right\}=\mathrm{Pr}\left\{\sum_{k=1}^{K}\log_{2}\left(1+\gamma_{E,k}\right)\geq R_{E}\right\},$$

where the accumulated mutual information of HARQ-IR after the *K*-th transmission is denoted by $I_{E}^{IR}\left(K\right)$. Assuming $Z_{E,K}=\prod_{k=1}^{K}Y_{E,k}$ and $Y_{E,k}=1+\gamma_{E,k}$ gives

(32) $$\mathrm{Pr}\left\{I_{E}^{IR}\left(K\right)\geq R_{E}\right\}=1-\mathrm{Pr}\left\{Z_{E,K}<2^{R_{E}}\right\}.$$

The above analysis suggests the *k*-th received SNR in the wiretap channel is an MG distributed RV with a PDF given by

(33) $$f_{\gamma_{E,k}}\left(\gamma \right)=\sum_{i=1}^{L}\alpha_{w,i}\gamma^{\beta_{w,i}-1}e^{-\zeta_{w,i}\gamma },\gamma \geq 0,$$

where $\alpha_{w,i}=\psi \left(\theta_{w,i},\beta_{w,i},\zeta_{w,i}\right)=\frac{\theta_{w,i}}{\sum_{j=1}^{L}\theta_{w,j}\Gamma \left(\beta_{w,j}\right)\zeta_{w,j}^{-\beta_{w,j}}}$, $\beta_{w,i}=m_{w}$, $\zeta_{w,i}=\frac{\lambda_{w}}{t_{i}}$, $\lambda_{w}={\tilde{\Xi }}_{w}^{2}$, $\theta_{w,i}=\frac{\lambda^{m_{w}}\omega_{i}t_{i}^{k_{w}-m_{w}-1}}{\Gamma \left(k_{w}\right)\Gamma \left(m_{w}\right)}$, $\omega_{i}$ and $t_{i}$ are given in [43], $k_{w}=L$, $m_{w}=1$ and ${\tilde{\Xi }}_{w}=\sqrt{\frac{1}{{\bar{\gamma }}_{E}}}$. Applying a similar approach in the main channel produces the CDF of $Z_{E,K}$ given by

(34) FZE,K(z)=∑r1⋯rLr1+⋯+rL=KKr1⋯rL∏j=1Lαw,jΓ(βw,j)ζw,j−βw,jrj×1−Y1,K+1K+1,0z∏j=1Lζw,jrj|(1,1,0,1)(0,1,0,1),(0,1,ζw,1,βw,1),⋯,(0,1,ζw,1,βw,1)︸r1items,⋯,(0,1,ζw,L,βw,L),⋯,(0,1,ζw,L,βw,L)︸rLitems

The SOP of HARQ-IR can then be obtained by

(35) $$\begin{array}{cc} P_{s}^{IR}\; & =\sum_{k=1}^{K}\mathrm{Pr}\left\{M=k\right\}\mathrm{Pr}\left\{I_{E}^{IR}\left(k\right)\geq R_{E}\right\}\\ \; & =\sum_{k=1}^{K}\left(F_{Z_{B,k-1}}\left(2^{R_{B}}-1\right)-F_{Z_{B,k}}\left(2^{R_{B}}-1\right)\right)\left(1-F_{Z_{E,k}}\left(2^{R_{E}}-1\right)\right), \end{array}$$

where $F_{Z_{B,k}}\left(z\right)$ and $F_{Z_{E,k}}\left(z\right)$ are provided in Equations (29) and (34), respectively.

## 4. Analysis of Diversity and Coding Gain

In this section, the asymptotic COP and SOP of both HARQ-CC and HARQ-IR are derived at higher SNR. The corresponding diversity and coding gain are then derived from these expressions, which reveal critical factors of secure performance.

### 4.1. Gain of IRS-Assisted Secure HARQ-CC

We first consider the diversity gain and coding gain of IRS-assisted secure HARQ-CC. Equations (12) and (14) can be used to reformulate the COP of HARQ-CC as

(36) $$\begin{array}{cc} P_{e}^{CC}\; & =F_{\Upsilon_{B,K}}\left(2^{R_{B}}-1\right)\\ \; & =\frac{1}{\Gamma \left(k_{M}\right)\Gamma \left(m_{M}\right)}G_{1,3}^{2,1}\left(\frac{\Xi_{M}^{2}\left(2^{R_{B}}-1\right)}{{\bar{\gamma }}_{B}}|\begin{array}{c} \kappa_{1}\\ \kappa_{2} \end{array}\right), \end{array}$$

where $\Xi_{M}=\sqrt{\frac{k_{M}m_{M}}{\Omega_{M}}}$, $\kappa_{1}=1$ and $\kappa_{2}=k_{M},m_{M},0$. Applying the asymptotic series expansion of Meijer’s G-function (provided in Appendix B) when ${\bar{\gamma }}_{B}\to \infty$ gives that the COP can be approximated as

(37) $$P_{e}^{CC}≃\frac{1}{\Gamma \left(k_{M}\right)\Gamma \left(m_{M}\right)}\sum_{k=1}^{2}{\left(\frac{{\bar{\gamma }}_{B}}{\Xi_{M}^{2}\left(2^{R_{B}}-1\right)}\right)}^{-\kappa_{2,k}}\frac{\prod_{l=1,l\neq k}^{2}\Gamma \left(\kappa_{2,l}-\kappa_{2,k}\right)\cdot \Gamma \left(\kappa_{2,k}\right)}{\Gamma (1+\kappa_{2,k})}.$$

It is worth noting that, if $\kappa_{2,l}=\kappa_{2,k}$, we can define $\Gamma \left(\kappa_{2,l}-\kappa_{2,k}\right)=\Gamma \left(\epsilon \right)$, where ϵ indicates a minor error term involved to meet the requirements of asymptotic series expansion. According to a definition in [46], when ${\bar{\gamma }}_{B}\to \infty$, $P_{e}^{CC}≃{\left(G_{c,m}^{CC}{\bar{\gamma }}_{B}\right)}^{-G_{d,m}^{CC}}$, where $G_{d,m}^{CC}$ and $G_{c,m}^{CC}$ represent the diversity gain and coding gain of the main channel in HARQ-CC. A comparison with Equation (37) gives

(38) $$G_{d,m}^{CC}=\min\left(k_{M},m_{M}\right)=K\min\left(k_{m},m_{m}\right).$$

Assuming $\kappa_{2,k^{*}}=\min\left(k_{m},m_{m}\right)$, where $k^{*}$ is the index of $\min\left(k_{m},m_{m}\right)$ in $\kappa_{2}$, gives

(39) $$\begin{array}{cc} G_{c,m}^{CC}\; & =\frac{1}{\Xi_{M}^{2}\left(2^{R_{B}}-1\right)}{\left(\frac{1}{\Gamma \left(k_{M}\right)\Gamma \left(m_{M}\right)}\frac{\prod_{l=1,l\neq k^{*}}^{2}\Gamma \left(\kappa_{2,l}-K\kappa_{2,k^{*}}\right)\cdot \Gamma \left(K\kappa_{2,k^{*}}\right)}{\Gamma (1+K\kappa_{2,k^{*}})}\right)}^{-\frac{1}{K\kappa_{2,k^{*}}}}\\ \; & =\frac{1}{\Xi_{M}^{2}\left(2^{R_{B}}-1\right)}{\left(\frac{1}{\Gamma \left(k_{M}\right)\Gamma \left(m_{M}\right)}\frac{\Gamma \left(K\left|k_{m}-m_{m}\right|\right)\cdot \Gamma \left(K\kappa_{2,k^{*}}\right)}{\Gamma (1+K\kappa_{2,k^{*}})}\right)}^{-\frac{1}{K\kappa_{2,k^{*}}}}. \end{array}$$

The diversity gain and coding gain in the wiretap channel of HARQ-CC can then be obtained by defining the connection outage probability of the wiretap channel as

(40) $$\begin{array}{cc} P_{e,w}^{CC} & =\mathrm{Pr}\left\{I_{E}^{CC}\left(\bar{M}\right)\leq R_{E}\right\}\\ \; & =F_{\Upsilon_{E,\bar{M}}}\left(2^{R_{E}}-1\right). \end{array}$$

Equation (19) then becomes

(41) $$P_{e,w}^{CC}=\frac{1}{\Gamma \left(\bar{M}N\right)\Gamma \left(\bar{M}\right)}G_{1,3}^{2,1}\left(\frac{\left(2^{R_{E}}-1\right)\bar{M}}{{\bar{\gamma }}_{E}}|\begin{array}{c} \kappa_{3}\\ \kappa_{4} \end{array}\right),$$

where $\kappa_{3}=1$ and $\kappa_{4}=\bar{M}N,\bar{M},0$. When ${\bar{\gamma }}_{B}\to \infty$ and ${\bar{\gamma }}_{E}\to \infty$, this implies $\bar{M}≃1$ and $\kappa_{4}≃N,1,0$. Applying a similar method in the main channel produces an outage probability of

(42) $$\begin{array}{c} P_{e,w}^{CC}≃\frac{1}{\Gamma (N)}\sum_{k=1}^{2}{\left(\frac{{\bar{\gamma }}_{E}}{2^{R_{E}}-1}\right)}^{-\kappa_{4,k}}\frac{\prod_{l=1,l\neq k}^{2}\Gamma \left(\kappa_{4,l}-\kappa_{4,k}\right)\cdot \Gamma \left(\kappa_{4,k}\right)}{\Gamma (1+\kappa_{4,k})}. \end{array}$$

The diversity gain and coding gain in the HARQ-CC wiretap channel can be denoted by $G_{d,w}^{CC}$ and $G_{c,w}^{CC}$, respectively, satisfying $P_{e,w}^{CC}≃{\left(G_{c,w}^{CC}{\bar{\gamma }}_{E}\right)}^{-G_{d,w}^{CC}}$. A comparison with Equation (42) suggests the diversity gain in the wiretap channel is

(43) $$G_{d,w}^{CC}=\min\left(N,1\right)=1.$$

While the coding gain is

(44) $$\begin{array}{cc} G_{c,w}^{CC} & =\frac{1}{2^{R_{E}}-1}{\left(\frac{1}{\Gamma (N)}\frac{\Gamma (N-1)\cdot \Gamma (1)}{\Gamma (2)}\right)}^{-1}\\ \; & =\frac{N-1}{2^{R_{E}}-1}. \end{array}$$

### 4.2. Gain of IRS-Assisted Secure HARQ-IR

We next consider the diversity gain and coding gain of IRS-assisted secure HARQ-IR. When ${\bar{\gamma }}_{B}\to \infty$, the COP of HARQ-IR can be represented as

(45) $$\begin{array}{cc} P_{e}^{IR}\; & =\mathrm{Pr}\left\{\sum_{k=1}^{K}\log_{2}\left(1+\gamma_{B,k}\right)<R_{B}\right\}\\ \; & ≃\mathrm{Pr}\left\{\log_{2}\prod_{k=1}^{K}\gamma_{B,k}<R_{B}\right\}\\ \; & =\mathrm{Pr}\left\{\prod_{k=1}^{K}\gamma_{B,k}<2^{R_{B}}\right\}, \end{array}$$

where $\gamma_{B,k}={\bar{\gamma }}_{B}R_{k}^{2}$ and $R_{k}$ denotes the signal magnitude of the *k*-th transmission. The PDF of $Y_{B,K}^{\prime }=\prod_{1}^{K}R_{k}$ can be determined using a moment generation function (MGF)-based method [47] by

(46) $$f_{Y_{B,K}^{\prime }}\left(y\right)=\frac{2}{y{\left(\Gamma \left(k_{m}\right)\Gamma \left(m_{m}\right)\right)}^{K}}G_{0,2K}^{2K,0}\left(y^{2}\Xi_{m}^{2K}|\begin{array}{c} -\\ \underset{2K\;\mathrm{items}}{\underset{︸}{k_{m},m_{m},\cdots ,k_{m},m_{m}}} \end{array}\right).$$

Defining $Z_{B,K}^{\prime }=\prod_{k=1}^{K}\gamma_{B,k}={\bar{\gamma }}_{B,k}Y_{B,K}^{\prime 2}$ then gives $f_{Z_{B,K}^{\prime }}\left(z\right)=f_{Y_{B,K}^{\prime }}\left(\sqrt{\frac{z}{{\bar{\gamma }}_{B}}}\right)\frac{1}{2\sqrt{{\bar{\gamma }}_{B}z}}$. In other words,

(47) $$\begin{array}{c} f_{Z_{B,K}^{\prime }}\left(z\right)=\frac{1}{z{\left(\Gamma \left(k_{m}\right)\Gamma \left(m_{m}\right)\right)}^{K}}G_{0,2K}^{2K,0}\left(z{\left(\frac{\Xi_{m}^{2}}{{\bar{\gamma }}_{B}}\right)}^{K}|\begin{array}{c} -\\ \underset{2K\;\mathrm{items}}{\underset{︸}{k_{m},m_{m},\cdots ,k_{m},m_{m}}} \end{array}\right). \end{array}$$

A corresponding integration implies the CDF of $Z_{K}^{\prime }$ is given by

(48) $$\begin{array}{c} F_{Z_{K}^{\prime }}\left(z\right)=\frac{1}{{\left(\Gamma \left(k_{m}\right)\Gamma \left(m_{m}\right)\right)}^{K}}G_{1,2K+1}^{2K,1}\left(z{\left(\frac{\Xi_{m}^{2}}{{\bar{\gamma }}_{B}}\right)}^{K}|\begin{array}{c} 1\\ \underset{2K\;\mathrm{items}}{\underset{︸}{k_{m},m_{m},\cdots ,k_{m},m_{m}}},0 \end{array}\right). \end{array}$$

Equation (45) suggests the COP of the main channel can be expressed as

(49) $$\begin{array}{cc} P_{e}^{IR} & =F_{Z_{K}^{\prime }}\left(2^{R_{B}}\right)\\ \; & =\frac{1}{{\left(\Gamma \left(k_{m}\right)\Gamma \left(m_{m}\right)\right)}^{K}}G_{1,2K+1}^{2K,1}\left(2^{R_{B}}{\left(\frac{\Xi_{m}^{2}}{{\bar{\gamma }}_{B}}\right)}^{K}|\begin{array}{c} \kappa_{5}\\ \kappa_{6} \end{array}\right), \end{array}$$

where $\kappa_{5}=1$ and $\kappa_{6}=\overset{k_{m},m_{m},\cdots ,k_{m},m_{m}}{\underset{2K\;\mathrm{items}}{︸}},0$. As stated in Appendix B, when ${\bar{\gamma }}_{B}\to \infty$,

(50) $$\begin{array}{c} P_{e}^{IR}=\frac{1}{{\left(\Gamma \left(k_{m}\right)\Gamma \left(m_{m}\right)\right)}^{K}}\sum_{k=1}^{2K}{\left(\frac{{\bar{\gamma }}_{B}}{2^{\frac{R_{B}}{K}}\Xi_{m}^{2}}\right)}^{-K\kappa_{6,k}}\frac{\prod_{l=1,l\neq k}^{2K}\Gamma \left(\kappa_{6,l}-\kappa_{6,k}\right)\cdot \Gamma \left(\kappa_{6,k}\right)}{\Gamma (1+\kappa_{6,k})}. \end{array}$$

The diversity gain and coding gain in the HARQ-IR main channel are denoted by $G_{d,m}^{IR}$ and $G_{c,m}^{IR}$, respectively, with the former expressed as

(51) $$G_{d,m}^{IR}=K\min\left(k_{w},m_{w}\right).$$

Assuming $\kappa_{6,k^{*}}=\min\left(k_{w},m_{w}\right)=\kappa_{2,k^{*}}$, the coding gain can be represented as

(52) $$\begin{array}{cc} G_{c,m}^{IR}= & \frac{1}{2^{\frac{R_{B}}{K}}\Xi_{m}^{2}}{\left(\frac{1}{{\left(\Gamma \left(k_{w}\right)\Gamma \left(m_{w}\right)\right)}^{K}}\frac{\prod_{l=1,l\neq k}^{2K}\Gamma \left(\kappa_{6,l}-\kappa_{6,k^{*}}\right)\cdot \Gamma \left(\kappa_{6,k^{*}}\right)}{\Gamma (1+\kappa_{6,k^{*}})}\right)}^{-\frac{1}{K\kappa_{6,k^{*}}}}\\ = & \frac{1}{2^{\frac{R_{B}}{K}}\Xi^{2}}{\left(\frac{1}{{\left(\Gamma \left(k_{w}\right)\Gamma \left(m_{w}\right)\right)}^{K}}\frac{\Gamma (\kappa_{6,k^{*}})}{\Gamma (1+\kappa_{6,k^{*}})}\left({\left(\Gamma (\left|k_{w}-m_{w}\right|\right)}^{K}{\left(\Gamma (\epsilon )\right)}^{K-1}\right)\right)}^{-\frac{1}{K\kappa_{6,k^{*}}}}. \end{array}$$

Since the actual transmission number of HARQ-IR is ${\bar{M}}^{\prime }=1$ when $\gamma_{B}\to \infty$, the same gain can be acquired as HARQ-CC. The diversity gain in the wiretap channel is then

(53) $$G_{d,w}^{IR}=\min\left(N,1\right)=1,$$

and the coding gain in the wiretap channel is

(54) $$\begin{array}{cc} G_{c,w}^{IR}\; & =\frac{1}{2^{R_{E}}-1}{\left(\frac{1}{\Gamma (N)}\frac{\Gamma (N-1)\cdot \Gamma (1)}{\Gamma (2)}\right)}^{-1}\\ \; & =\frac{N-1}{2^{R_{E}}-1}. \end{array}$$

## 5. Numerical Results

In this section, through Monte Carlo simulations and numerical results obtained from the analytical expressions above, we analyze the outage probability performance of IRS assisted secure HARQ, using the model shown in Figure 1. The main secure performance metrics, including COP, SOP and their asymptotic value of HARQ-CC and HARQ-IR, are primarily discussed with a given constraint of maximum transmission number.

**Figure 2:**
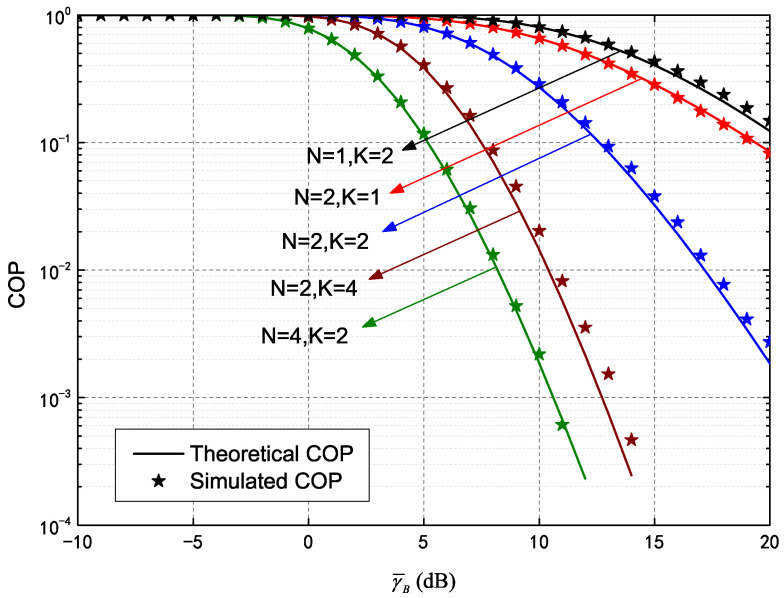
Connection outage probability (COP) of HARQ-CC versus ${\bar{\gamma }}_{B}$ for different *N* and *K*.

Figure 2 shows COP curves plotted against ${\bar{\gamma }}_{B}$ of a secure HARQ-CC system assisted by an IRS, demonstrating the influence of different re-transmission numbers and meta-surfaces. The parameters were set as ${\bar{\gamma }}_{B}=-10∼20$ dB, $R_{B}=5$, $R_{E}=2$, with five pairs of curves configured as different combinations of K=1,2,4 and N=1,2,4. The solid lines and asterisks represent the theoretical and simulated COP, respectively. It can be observed that the differences between these two curves are trivial, verifying the theoretical accuracy of Equations (12) and (14). In addition, the COP decreases monotonically with the increase of ${\bar{\gamma }}_{B}$, as higher quality of the main channel resulted in fewer connection outages. The COP also decreased significantly with increasing *K*, due to an accumulated SNR for HARQ-CC. The increase in *N* allowed the IRS to provide more channels through meta-surfaces, thereby reducing connection outage. Furthermore, it is also evident that increasing *N* had more of an effect on the COP of HARQ-CC than increasing *K* (e.g., the results of N=2,K=1 outperforms N=1,K=2 and N=4,K=2 outperforms N=2,K=4). This is because chase combining adopts simple repeated transmission with a limited gain.

**Figure 3:**
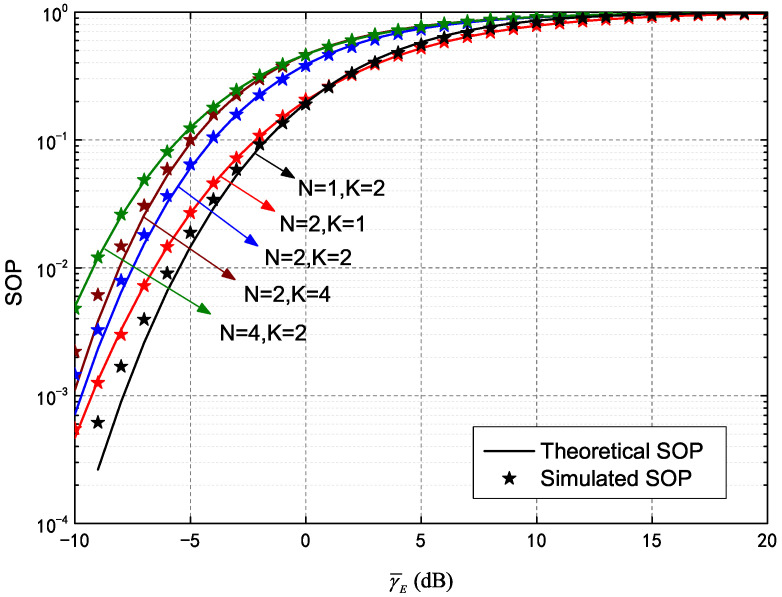
Secrecy outage probability (SOP) of HARQ-CC versus ${\bar{\gamma }}_{E}$ for different *N* and *K*.

Figure 3, which depicts SOP performance of an IRS-assisted HARQ-CC system, demonstrates the influence of different transmission numbers and meta-surface quantities. SOP curves were plotted against different values of ${\bar{\gamma }}_{E}$, including ${\bar{\gamma }}_{B}=10$ dB, ${\bar{\gamma }}_{E}=-10∼20$ dB, $R_{B}=5$, $R_{E}=2$ and five additional pairs of curves configured as given in Figure 2. It is evident the theoretical SOP (solid line) values agree well with the simulated results (asterisks). In addition, increases in ${\bar{\gamma }}_{E}$ are strongly correlated with an increase in SOP, which indicates that higher quality in the wiretap channel worsens secrecy outage. Furthermore, increases in *K* were seen to cause SOP increases, though the increment of N=2,K=4 (relative to N=2,K=2) was less than that of N=2,K=2 (relative to N=2,K=1). This is because the COP is higher during the first transmission than at the second round. In other words, more re-transmissions occur for lower gain in the wiretap channel. In addition, the SOP deteriorates significantly with increasing *N* (e.g., the results for N=4,K=2 are higher than for N=2,K=2). This is caused by more assisted channels implemented through meta-surfaces for both the main channel and wiretap channel.

**Figure 4:**
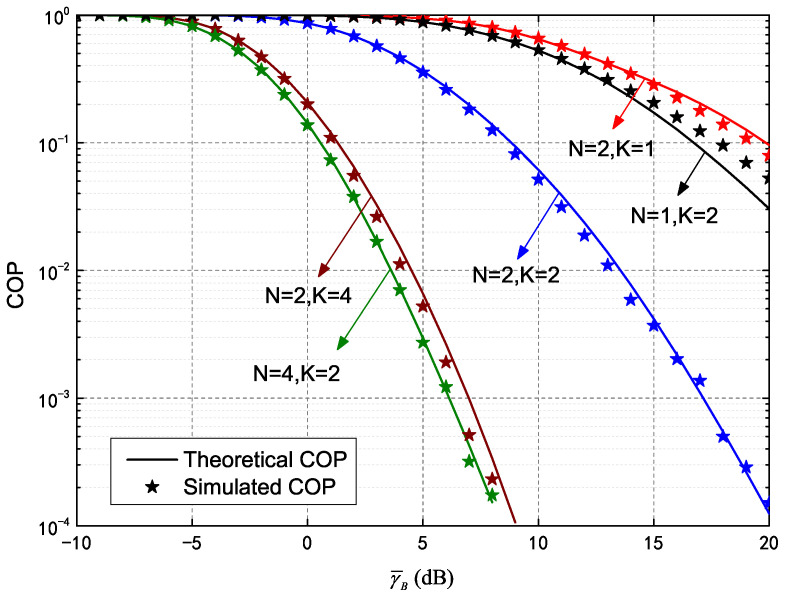
Connection outage probability (COP) of HARQ-IR versus ${\bar{\gamma }}_{B}$ for different *N* and *K*.

**Figure 5:**
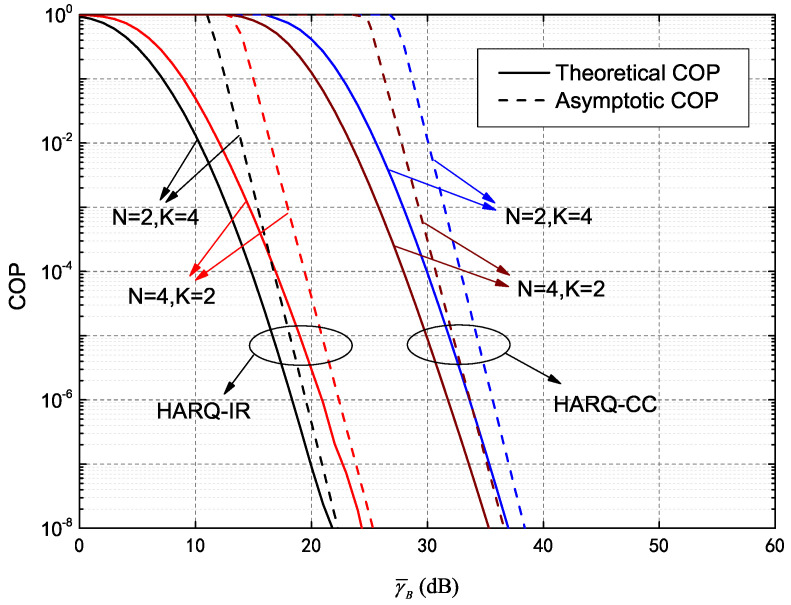
Asymptotic COP versus ${\bar{\gamma }}_{B}$ of both HARQ-CC and HARQ-IR for different *N* and *K*.

Figure 4 plots COP versus ${\bar{\gamma }}_{B}$ of a secure HARQ-IR system assisted by IRS, demonstrating the influence of different re-transmission and meta-surface quantities. Parameters in the figure were set as in Figure 2. This analysis of the COP was also verified by theoretical and simulated results, denoted by the solid lines and asterisks, respectively. The COP in Equation (29) decreases monotonically as ${\bar{\gamma }}_{B}$ increases, indicating higher quality in the main channel with fewer connection outages. The COP decreased significantly as *K* increased, indicating the coding gain in HARQ-IR can be determined for better connection performance. The IRS then provides more channels as *N* increases, thereby reducing the COP. It is worth noting the effects of increasing *N* and increasing *K* differed for HARQ-IR under various conditions. For instance, the COP for N=1,K=2 outperformed that of N=2,K=1 and N=2,K=4 outperformed N=4,K=2. As such, the maximum transmission number should be increased at higher SNR, while meta-surface quantities should be increased at lower SNR. This is because connections benefit more from channel diversity than from coding in the case of lower SNRs and vice versa, as verified in Figure 5.

COP curves of the HARQ system assisted by IRS are plotted with their asymptotic values in Figure 5, for a case where ${\bar{\gamma }}_{B}$ is large. Parameters were set as ${\bar{\gamma }}_{B}=0∼60$ dB, $R_{B}=10$ and $R_{E}=5$. The solid and dotted lines represent theoretical and asymptotic HARQ-CC and HARQ-IR COP values for N=2,K=4 and N=4,K=2, respectively. The simulated values have already been verified and are not repeated here for clarity. It is evident that when ${\bar{\gamma }}_{B}\to \infty$, each theoretical COP value comes first at lower SNR and tends to an asymptotic limit. The COP of N=4,K=2 is then better than that of N=2,K=4 when HARQ-CC is adopted. Thus, the gain resulting from an increase in the number of meta-surfaces is larger than that caused by increasing the maximum transmission number, which is consistent with our analysis from Figure 2. Furthermore, the COP for N=2,K=4 is superior to that of N=4,K=2 when HARQ-IR is adopted. In other words, increasing the maximum transmission number is more effective than increasing the number of meta-surfaces, which is concordant with the high-SNR results depicted in Figure 4.

**Figure 6:**
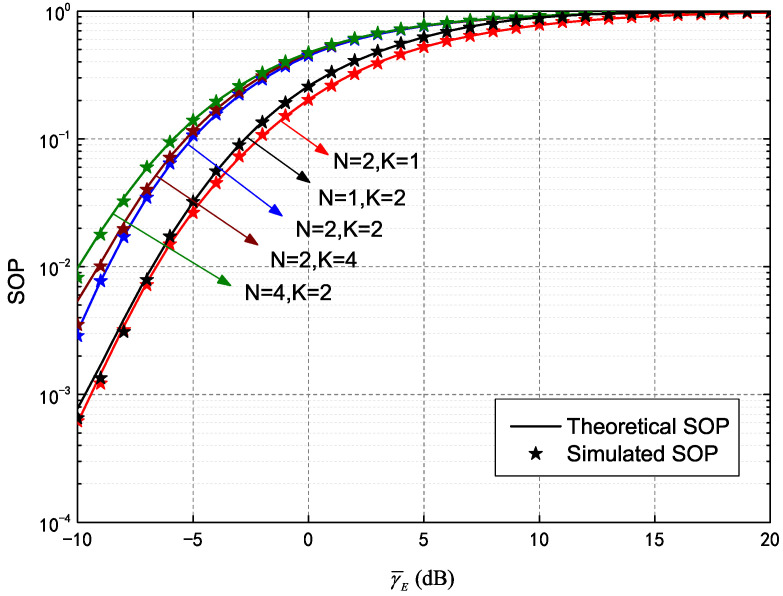
Secrecy outage probability (SOP) of HARQ-IR versus ${\bar{\gamma }}_{E}$ for different *N* and *K*.

Figure 6 plots SOP performance of an IRS-assisted HARQ-IR system as a function of ${\bar{\gamma }}_{E}$, *N* and *K*. The simulated parameters are the same as those of the SOP simulation for HARQ-CC as shown in Figure 3. The difference between theoretical and simulated results is slight, while the former is given in Equation (35). As shown, the SOP continues to increase with ${\bar{\gamma }}_{E}$ and *K*, though the increment of N=2,K=4 relative to N=2,K=2 is lower than that of N=2,K=2 relative to N=2,K=1. The observed gain in the wiretap channel also decreased more sharply than that of HARQ-CC, due to stronger coding gain for HARQ-IR. In addition, the SOP further increased as *N* increased, though the increment decreased with increasing *N*. For instance, the SOP of N=2,K=2 increased significantly relative to N=1,K=2, while that of N=4,K=2 changed little compared to N=2,K=2. This was due to a decrease in COP, a decrease in the actual transmission number and corresponding limited IRS-assisted gain.

**Figure 7:**
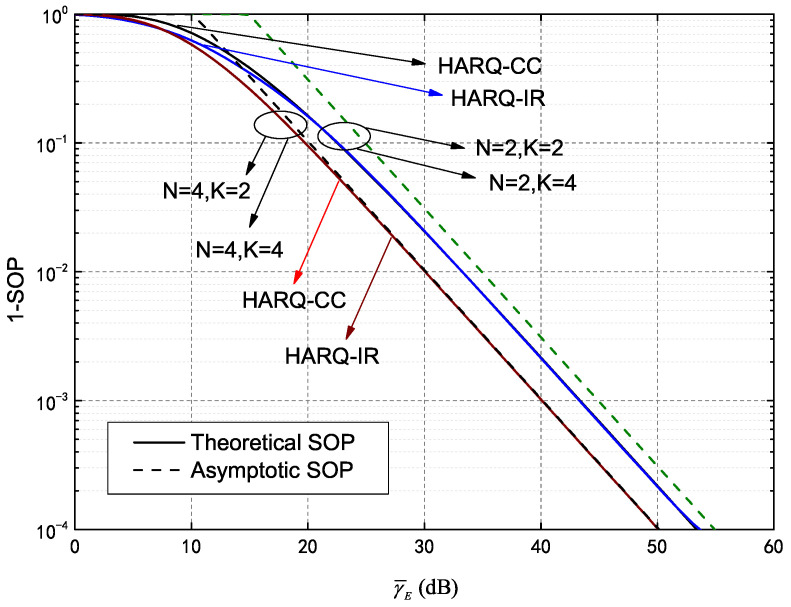
Asymptotic 1-SOP versus ${\bar{\gamma }}_{E}$ of both HARQ-CC and HARQ-IR for different *N* and *K*.

Figure 7 provides an asymptotic SOP analysis of the IRS-assisted HARQ system. Curves are included for the 1-SOP and its asymptotic values in HARQ-CC and HARQ-IR for increased clarity. Among these curves, ${\bar{\gamma }}_{B}=30$ dB, ${\bar{\gamma }}_{E}=0∼60$ dB and all other parameters are the same as in Figure 5. The solid and dashed lines represent the theoretical and asymptotic SOP values, respectively. Each 1-SOP theoretical value tends to an asymptotic limit when ${\bar{\gamma }}_{E}\to \infty$, which supports our presented analysis. In addition, the 1-SOP curves for HARQ-CC and HARQ-IR tended to be consistent for a given *N* configuration, such as N=2,K=2 and N=2,K=4 or N=4,K=2 and N=4,K=4. The asymptotic SOP values were also the same, which further verifies our analysis of SOP (i.e., the asymptotic values are only dependent on *N* with given $R_{E}$).

## 6. Conclusions

In this paper, we derived closed-form COP and SOP expressions of HARQ-CC and HARQ-IR using the KG distribution, the MG distribution and the Mellin transform. The asymptotic value analysis of the COP and SOP was performed to determine the diversity and coding gain. An analysis of numerical and simulated results suggested that expanding the number of meta-surfaces had a significant impact on the main channel for all SNR regions in HARQ-CC and lower-SNR regions in HARQ-IR while increasing the maximum transmission number critically affected the main channel for higher-SNR regions in HARQ-IR. Additionally, only the number of meta-surfaces mainly affected the wiretap channel for both HARQ-CC and HARQ-IR. On this basis, corresponding strategies could be further designed to effectively improve physical layer security performance for an IRS-assisted HARQ system.

## Appendix A. The Inverse Mellin Transform of $M_{s}\left\{f_{Z_{B,K}}\left(z\right)\right\}$

Here, we obtain the inverse Mellin transform of $M_{s}\left\{f_{Z_{B,K}}\left(z\right)\right\}$ as follows,

(A1) $$\begin{array}{cc} f_{Z_{B,K}}\left(z\right)= & M_{s}^{-1}\left[M_{s}\left\{f_{Z_{B,K}}\left(z\right)\right\}\right]\\ = & \frac{1}{2\pi i}\int_{c-i\infty }^{c+i\infty }M_{s}\left\{f_{Z_{B,K}}\left(z\right)\right\}z^{-s}ds. \end{array}$$

Substituting Equation (27) into Equation (A1) gives

(A2) fZB,K(z)=12πi∫c−i∞c+i∞∑r1⋯rLr1+⋯+rL=KKr1⋯rL∏j=1Lαm,jΓ(βm,j)Ψ(βm,j,βm,j+s;ζm,j)rjz−sds=∑r1⋯rLr1+⋯+rL=KKr1⋯rL∏j=1Lαm,jΓ(βm,j)rj12πi∫c−i∞c+i∞∏j=1LΨ(βm,j,βm,j+s;ζm,j)rjz−sds

For convenience of presentation, we define

(A3) $$I_{0}=\frac{1}{2\pi i}\int_{c-i\infty }^{c+i\infty }\prod_{j=1}^{L}{\left(\Psi (\beta_{m,j},\beta_{m,j}+s;\zeta_{m,j})\right)}^{r_{j}}z^{-s}ds.$$

An expression of $I_{0}$ is provided as

(A4) $$\begin{array}{c} I_{0}=\prod_{j=1}^{L}{\left(\zeta_{m,j}^{\beta_{m,j}-1}\right)}^{-r_{j}}Y_{0,K}^{K,0}\left(z\prod_{j=1}^{L}\zeta_{m,j}^{r_{j}}|\begin{array}{c} -\\ \underset{r_{1}\;\mathrm{items}}{\underset{︸}{\left(0,1,\zeta_{m,1},\beta_{m,1}\right),\cdots ,\left(0,1,\zeta_{m,1},\beta_{m,1}\right)}},\cdots ,\underset{r_{L}\;\mathrm{items}}{\underset{︸}{\left(0,1,\zeta_{m,L},\beta_{m,L}\right),\cdots ,\left(0,1,\zeta_{m,L},\beta_{m,L}\right)}} \end{array}\right) \end{array}$$

where $Y_{p,q}^{m,n}\left[r\right]$ denotes the generalized Fox’s H function which is defined as

(A5) $$\begin{array}{c} Y_{p,q}^{m,n}\left(r|\begin{array}{c} \left(a_{1},\alpha_{1},A_{1},\varphi_{1}\right),\cdots ,\left(a_{p},\alpha_{p},A_{p},\varphi_{p}\right)\\ \left(b_{1},\beta_{1},B_{1},\phi_{1}\right),\cdots ,\left(b_{q},\beta_{q},B_{q},\phi_{q}\right) \end{array}\right)=\frac{1}{2\pi i}\int_{L}M_{p,q}^{m,n}\left[s\right]x^{-s}ds, \end{array}$$

where L is the Mellin-Barnes contour of the complex s plane from c−i∞ to c+i∞ and c∈R. The term $M_{p,q}^{m,n}\left[s\right]$ is defined as

(A6) $$\begin{array}{c} M_{p,q}^{m,n}\left[s\right]=\frac{\prod_{j=1}^{m}B_{j}^{\phi_{j}+b_{j}+\beta_{j}s-1}\Psi \left(\phi_{j},\phi_{j}+b_{j}+\beta_{j}s;B_{j}\right)}{\prod_{i=n+1}^{p}A_{i}^{\varphi_{i}+a_{i}+a_{i}s-1}\Psi \left(\varphi_{i},\varphi_{i}+a_{i}+\alpha_{i}s;A_{i}\right)}\frac{\prod_{i=1}^{n}A_{i}^{\varphi_{i}-a_{i}-a_{i}s}\Psi \left(\varphi_{i},\varphi_{i}+1-a_{i}-\alpha_{i}s;A_{i}\right)}{\prod_{j=m+1}B_{j}^{\phi_{j}-b_{j}-\beta_{j}s}\Psi \left(\phi_{j},\phi_{j}+1-b_{j}-\beta_{j}s;B_{j}\right)} \end{array}$$

Inserting $I_{0}$ into Equation (A2) produces the expression of $f_{Z_{B,K}}\left(z\right)$ shown in Equation (28).

## Appendix B. The Asymptotic Series Expansion of Meijer’s G-Function at z→0

The asymptotic series expansion of Meijer’s G-function at z→0 is given by [48]

(A7) $$\begin{array}{c} G_{p,q}^{m,n}\left(z|\begin{array}{c} a_{1},\cdots ,a_{n},a_{n+1},\cdots ,a_{p}\\ b_{1},\cdots ,b_{m},b_{m+1},\cdots ,b_{q} \end{array}\right)≃\sum_{k=1}^{m}z^{b_{k}}\frac{\prod_{j=1,j\neq k}^{m}\Gamma \left(b_{j}-b_{k}\right)\prod_{j=1}^{n}\Gamma \left(1-a_{j}+b_{k}\right)}{\prod_{j=n+1}^{p}\Gamma \left(a_{j}-b_{k}\right)\prod_{j=m+1}^{q}\Gamma \left(1-b_{j}+b_{k}\right)}, \end{array}$$

with j∉k and $b_{j}-b_{k}\notin Z$.

## References

[1] Wu Q., Zhang R. (2020). Towards Smart and Reconfigurable Environment: Intelligent Reflecting Surface Aided Wireless Network. IEEE Commun. Mag..

[2] Di Renzo M., Zappone A., Debbah M., Alouini M.-S., Yuen C., De Rosny J., Tretyakov S. (2020). Smart Radio Environments Empowered by Reconfigurable Intelligent Surfaces: How It Works, State of Research, and The Road Ahead. IEEE J. Sel. Areas Commun..

[3] Yu X., Xu D., Schober R. Enabling secure wireless communications via intelligent reflecting surfaces. In Proceeding of the IEEE Global Communications Conference (GLOBECOM).

[4] Hong S., Pan C., Ren H., Chai K., Nallanathan A. (2021). Robust transmission design for intelligent reflecting surface-aided secure communication systems with imperfect cascaded CSI. IEEE Trans. Wirel. Commun..

[5] Zhou G., Pan C., Ren H., Wang K., Nallanathan A. (2020). Framework of Robust Transmission Design for IRS-Aided MISO Communications with Imperfect Cascaded Channels. IEEE Trans. Signal Process..

[6] Barar E., Di Renzo M., De Rosny J., Debbah M., Alouini M.-S., Zhang R. (2019). Wireless communications through reconfigurable intelligent surfaces. IEEE Access.

[7] Yang L., Meng F., Wu Q., Da Costa D., Alouini M.-S. (2020). Accurate closed-form approximations to channel distributions of RIS-aided wireless systems. IEEE Wirel. Commun. Lett..

[8] Zhang H., Di B., Song L., Han Z. (2020). Reconfigurable intelligent surfaces assisted communications with limited phase shifts: How many phase shifts are enough?. IEEE Trans. Veh. Technol..

[9] Atapattu S., Fan R., Dharmawansa P., Wang G., Evans J., Tsiftsis T.A. (2020). Reconfigurable intelligent surface assisted two–way communications: Performance analysis and optimization. IEEE Trans. Commun..

[10] Boulogeorgos A.-A.A., Alexiou A. (2020). Performance analysis of reconfigurable intelligent surface-assisted wireless systems and comparison with relaying. IEEE Access.

[11] Huang C., Zappone A., Alexandropoulos G.C., Debbah M., Yuen C. (2019). Reconfigurable intelligent surfaces for energy efficiency in wireless communication. IEEE Trans. Wirel. Commun..

[12] Guo H., Liang Y.C., Chen J., Larsson E.G. (2020). Weighted sum-rate maximization for reconfigurable intelligent surface aided wireless networks. IEEE Trans. Wirel. Commun..

[13] Yang Y., Zhang S., Zhang R. (2020). IRS-enhanced OFDMA: Joint resource allocation and passive beamforming optimization. IEEE Commun. Lett..

[14] Wang Y., Yin H., Zhang T., Yang W., Shang X., Shen Y. (2022). Secure Transmission for Energy-Harvesting Sensor Networks with a Buffer-Aided Sink Node. IEEE Internet Things J..

[15] Diao D., Wang B., Cao K., Dong R., Cheng T. (2022). Enhancing Reliability and Security of UAV-Enabled NOMA Communications with Power Allocation and Aerial Jamming. IEEE Trans. Veh. Technol..

[16] Yang L., Yang J., Xie W., Hasna M.O., Tsiftsis T., Di Renzo M. (2020). Secrecy performance analysis of RIS-aided wireless communication systems. IEEE Trans. Veh. Technol..

[17] Khoshafa M.H., Ngatched T.M.N., Ahmed M.H. (2020). Reconfigurable intelligent surfaces-aided physical layer security enhancement in D2D underlay communications. IEEE Commun. Lett..

[18] Shen H., Xu W., Gong S., He Z., Zhao C. (2019). Secrecy rate maximization for intelligent reflecting surface assisted multi-antenna communications. IEEE Commun. Lett..

[19] Cui M., Zhang G., Zhang R. (2019). Secure wireless communication via intelligent reflecting surface. IEEE Commun. Lett..

[20] Hong S., Pan C., Ren H., Wang K., Nallanathan A. (2020). Artificial-noise-aided secure MIMO wireless communications via intelligent reflecting surface. IEEE Trans. Commun..

[21] Tsoulos I.G., Stavrou V., Mastorakis N.E., Tsalikakis D. (2019). GenConstraint: A programming tool for constraint optimization problems. SoftwareX.

[22] Yu X., Xu D., Sun Y., Ng D.W.K., Schobe R. (2020). Robust and Secure Wireless Communications via Intelligent Reflecting Surfaces. IEEE J. Sel. Areas Commun..

[23] Wang H.M., Bai J., Dong L. (2020). Intelligent Reflecting Surfaces Assisted Secure Transmission without Eavesdropper’s CSI. IEEE Signal Process. Lett..

[24] Gu X., Duan W., Zhang G., Sun Q., Wen M., Ho P.H. (2022). Physical Layer Security for RIS-Aided Wireless Communications with Uncertain Eavesdropper Distributions. IEEE Syst. J..

[25] Chelli A., Zedini E., Alouini M.-S., Barry J.R., Patzold M. (2014). Performance and Delay Analysis of Hybrid ARQ with Incremental Redundancy Over Double Rayleigh Fading Channels. IEEE Trans. Wirel. Commun..

[26] Shi Z., Ma S., Yang G., Tam K.-W., Xia M. (2017). Asymptotic Outage Analysis of HARQ-IR Over Time-Correlated Nakagami- *m* Fading Channels. IEEE Trans. Wirel. Commun..

[27] Zedini E., Chelli A., Alouini M.-S. (2014). On the Performance Analysis of Hybrid ARQ with Incremental Redundancy and with Code Combining Over Free-Space Optical Channels with Pointing Errors. IEEE Photon. J..

[28] Tang X., Liu R., Spasojevic P., Poor H.V. (2009). On the throughput of secure hybrid-ARQ protocols for Gaussian block-fading channels. IEEE Trans. Inf. Theory.

[29] Mheich Z., Treust M.L., Alberge F., Duhamel P. (2016). Rate Adaptation for Incremental Redundancy Secure HARQ. IEEE Trans. Commun..

[30] Guan X., Cai Y., Yang W. (2014). On the Reliability-Security Tradeoff and Secrecy Throughput in Cooperative ARQ. IEEE Commun. Lett..

[31] Wu Y., Yin S., Zhou J., Yang P., Yang H. (2020). Quasi-concave optimization of secrecy redundancy rate in HARQ-CC system. Sci. China Inf. Sci..

[32] Wu Y., Yin S., Zhou J., Yang P., Yang H. (2020). Rate Adaption for Secure HARQ-CC System with Multiple Eavesdroppers. Entropy.

[33] Park S. (2023). Kalman Combining Based Iterative Detection and Decoding for MIMO Systems with Hybrid ARQ. IEEE Trans. Veh. Technol..

[34] Wu S., Deng Z., Li A., Jiao J., Zhang N., Zhang Q. (2023). Minimizing Age-of-Information in HARQ-CC Aided NOMA Systems. IEEE Trans. Wirel. Commun..

[35] Cao Q., Zhang H., Wang H., Fu Y., Yang G., Ma S. Outage Performance Analysis of HARQ-Aided Multi-RIS Systems. In Proceeding of IEEE Wireless Communications and Networking Conference (WCNC).

[36] Gradshteyn I.S., Ryzhik I.M. (2000). Table of Integrals, Series, and Products.

[37] Wyner A.D. (1975). The wire-tap channel. Bell. Sys. Tech. J..

[38] Bjornson E., Sanguinetti L. (2020). Power Scaling Laws and Near-Field Behaviors of Massive MIMO and Intelligent Reflecting Surfaces. IEEE Open J. Commun. Soc..

[39] Peppas K.P. (2011). Accurate closed-form approximations to generalised-K sum distributions and applications in the performance analysis of equal-gain combining receivers. IET Commun..

[40] Liu H., Ding H., Xiang L., Yuan J., Zhang L. (2014). Outage and BER Performance Analysis of Cascade Channel in Relay Networks. Procedia Comput. Sci..

[41] Chatzidiamantis N.D., Karagiannidis G.K. (2011). On the Distribution of the Sum of Gamma-Gamma Variates and Applications in RF and Optical Wireless Communications. IEEE Trans. Commun..

[42] Atapattus S., Tellambura C., Jiang H. (2011). A Mixture Gamma Distribution to Model the SNR of Wireless Channels. IEEE Trans. Wirel. Commun..

[43] Abramowitz M., Stegun I.A. (1965). Handbook of Mathematical Functions: With Formulas, Graphs, and Mathematical Tables.

[44] Debnath L., Bhatta D. (2010). Integral Transforms and Their Applications.

[45] Yilmaz F., Alouini M.-S. Outage capacity of multicarrier systems. In Proceeding of the IEEE International Conference on Telecommunications (ICT).

[46] Wang Z., Giannakis G.B. (2003). A simple and general parameterization quantifying performance in fading channels. IEEE Trans. Commun..

[47] Trigui I., Laourine A., Affes S., Stephenne A. On the performance of cascaded generalized k fading channels. In Proceeding of the IEEE Global Telecommunications Conference (GLOBECOM).

[48] Wolfram Research I. (2010). Mathematica Edition: Version 8.0.

